# Function of Nuclear Pore Complexes in Regulation of Plant Defense Signaling

**DOI:** 10.3390/ijms23063031

**Published:** 2022-03-11

**Authors:** Xi Wu, Junyou Han, Changkui Guo

**Affiliations:** 1 Jilin Province Engineering Laboratory of Plant Genetic Improvement, College of Plant Science, Jilin University, Changchun 130062, China; qianwu18@mails.jlu.edu.cn; 2 Laboratory of Plant Molecular and Developmental Biology, Zhejiang A & F University, Hangzhou 311300, China

**Keywords:** nuclear pore complex, nucleoporin, structure, biological function, defense signaling

## Abstract

In eukaryotes, the nucleus is the regulatory center of cytogenetics and metabolism, and it is critical for fundamental biological processes, including DNA replication and transcription, protein synthesis, and biological macromolecule transportation. The eukaryotic nucleus is surrounded by a lipid bilayer called the nuclear envelope (NE), which creates a microenvironment for sophisticated cellular processes. The NE is perforated by the nuclear pore complex (NPC), which is the channel for biological macromolecule bi-directional transport between the nucleus and cytoplasm. It is well known that NPC is the spatial designer of the genome and the manager of genomic function. Moreover, the NPC is considered to be a platform for the continual adaptation and evolution of eukaryotes. So far, a number of nucleoporins required for plant-defense processes have been identified. Here, we first provide an overview of NPC organization in plants, and then discuss recent findings in the plant NPC to elaborate on and dissect the distinct defensive functions of different NPC subcomponents in plant immune defense, growth and development, hormone signaling, and temperature response. Nucleoporins located in different components of NPC have their unique functions, and the link between the NPC and nucleocytoplasmic trafficking promotes crosstalk of different defense signals in plants. It is necessary to explore appropriate components of the NPC as potential targets for the breeding of high-quality and broad spectrum resistance crop varieties.

## 1. Introduction

The nuclear envelope (NE) is the key distinguishing factor between eukaryotes and prokaryotes, and it compartmentalizes a microenvironment for dedicated biological processes including genomic DNA replication and repair, transcription, and RNA processing. The NE is embedded with numerous small holes called nuclear pores, through which the largest and sophisticated protein complex, the nuclear pore complex (NPC), penetrates. The NPC mediates the selective transport of macromolecules, and allows for the passive transport of molecular cargos smaller than 40 kDa. Activating the transport of larger molecules requires the assistance of nuclear transport receptors (NTRs) [1,2].

The NPC is composed of approximately 30 kinds of NUPs [1,2]. Different NUPs are arranged in distinct NPC subcomplexes and perform unique cellular processes [1]. In addition to their basic function in facilitating the transport of biomolecules between the nucleus and cytoplasm [3], NPC also function in regulating the spatial location of chromatin and RNA processing in a transport-independent manner [4,5,6,7]. The spatial segregation of euchromatin and heterochromatin in nuclei is not a random event [8,9]. The research of microscopy technology combined with high resolution chromosome conformation capture (Hi-C) indicates that euchromatin localized in the nuclear interior and heterochromatin is primarily positioned near the nuclear periphery, whereas the phase separation induced by interactions of the heterochromatin play central roles in establishing compartmentalization [10]. Recent evidence shows that transcription factors (taken Gcn4 as an example) can recruit target genes and tether them to NPC for controlling the spatial arrangement of the yeast genome [11]. Moreover, NPC can regulate the chromatin condensation state by interacting with Poly-comb Repressive Complex, nucleosome, and histone-modifying enzymes [7]. Therefore, future works may focus on the function of NPC in controlling gene activation and spatial organization in plant cells independent traffic roles [12].

Previous studies of NPC mostly focus on the structure and function of metazoan cell or yeast. However, NPCs were poorly understood before the application of advanced biology approaches, such as bioinformatics, atomic force microscopy (AFM), crystallography [13,14]. In the past, the forms of different NPCs were considered to be identical, but several recent studies have uncovered that the expression of NPC components varies in different cell types and different nucleoporins can play precise roles in the development and homeostasis of specific organs [15]. Cryo-EM combined with 3D modeling technology not only uncover novel perspectives in NPC architecture, also reveal that distinct NPC forms (at least three) co-existing in the same cell [16,17]; these distinct forms may be assembled to respond to a particular biological signal.

Our understanding of plant NPC is still far from complete. In our review, we summarize the recent studies of the microstructure and biological functions of plant NPC, and emphasize the biological functions of NPC in the immune defense, growth and development, hormone signaling, and temperature response in plants. We also review the recent findings for regulatory mechanisms by which NPC is involved in plant defensive signaling processes, and compare regulation mechanisms between plants and other organisms.

## 2. Sketch of Plant NPC

When investigating the functions of the NPC components participating in defensive signaling pathways in plants, the structural composition of plant NPC should be outlined first. Due to their small size and structural plasticity, identifying the microstructures of plant NPC has been a particularly difficult challenge [18]. Continuous advances in research technologies, such as proteomics, crystallography, bioinformatics, and cryo-electron tomography (cryo-ET) [19] have aroused gradually distinct understandings of NPC microstructures in plants [20,21,22,23]. Recently, scientists use a buffer with a high sucrose concentration to purify nuclei and then conduct solubility-based fractionation to increase proteome coverage; finally, they identify two novel nucleoporins (At1g07970 and At3g08870). Interestingly, At3g08879 was first reported as a kinase domain containing protein localized on the NE in plants [24]. However, our understanding of NPC microstructures in plant and their exact components remains incomplete.

**Figure 1:**
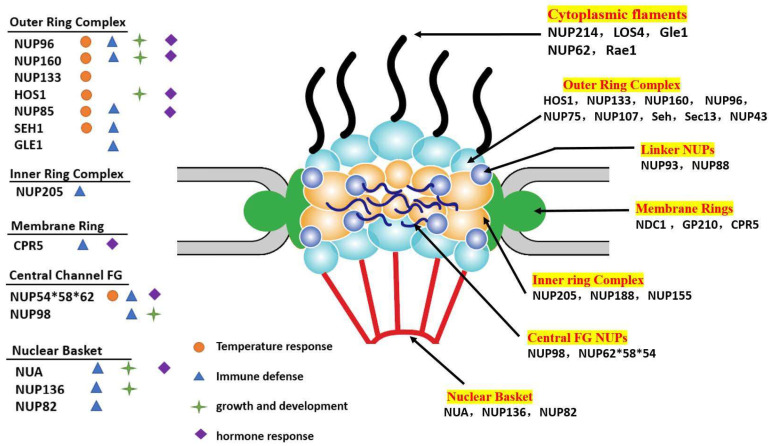
A model for the plant NPC structural organization and the role of plant NUPs in different biological processes. The NPCs consist of the following main components: scaffold nucleoporins include outer ring (blue), inner ring (yellow), the transmembrane ring nucleoporins (green), linker nucleoporins (purple), cytoplasmic filaments (dark) and nuclear basket (red), and central FG nucleoporins (dark purple). NUPs that have been functionally investigated in plants are underlined; and the left part lists some plant NUPs that have been reported to be involved in immune signaling (blue triangle), growth and development (green star), hormone response (purple diamond), and temperature response (orange cycle).

In general, NPC has a diameter of 100–150 nm and a depth of 50–70 nm, and is composed of a ring that consists of eight units arranged in radial symmetry [25]. The NPC comprises several conserved modules that can be divided into several subcomplexes, such as the core scaffold, cytoplasmic filaments, nuclear basket, membrane ring, and central FG (Phe-Gly) nucleoporins (that contain intrinsically disordered phenylalanine glycine) (Figure 1) [4].

### 2.1. Core Scaffold

The core scaffold nucleoporins are the most conserved and flexible among the different NPC modules. The significant flexibility of core scaffold nucleoporins may promote their interaction with specific assemblies such as silencing factor Sir4, histone deacetylase HDAC4 and heterochromatin [26,27,28,29].The function and structure of the core scaffold has been widely studied in yeasts [30], thermophilic fungi [31] and toad [32]. However, the core scaffold remains to be understood in plants. The core scaffold consists of two subcomplexes: the outer ring complex (ORC) and the inner ring complex (IRC).

The ORC is formed by connecting the head to the tail in Y-shaped complexes, which are assembled by 6–10 highly conserved NUPs (the specific number depends on the species), including NUP160, NUP 107, NUP96, NUP133, NUP85, NUP43, NUP37, SEH1, and SEC13 [4]. Eight Y-shaped complexes assemble a ring with an outer diameter of around 125 nm and inner diameter of around 50 nm by connecting the head and tail [32,33]. However, the copies of Y-complex in each ORC are not consistent from species to species. A study describes that the Y-complex of fission yeast is split into two pieces and asymmetrically located at the nuclear and cytoplasmic respective. This asymmetric form of Y-complex is necessary for cell-cycle progression in yeast [34]. Another recent study found that the core scaffold of the early branching eukaryote *Chlamydomonas reinhardtii* (algal) exhibits differences between human and yeast core scaffolds [35]. First, the algal IRC diameter is 64 nm, and the human IRC diameter is 43 nm, which can facilitate larger molecule trafficking across the algal NPC. Second, the algal ORC is in an asymmetric ‘hybrid’ state, with the nucleus side in a bi-layered ring structure assembled by 16 Y-complexes, with the cytoplasmic side being single-layered (8 Y-complexes). This unique configuration has never been observed in other species, whereas the yeast ORC is in a single-layered state and human ORC is double layered. This asymmetric hybrid configuration indicates an intermediate evolutionary pattern in early branching eukaryotes. This study investigates the variation in the plant nucleocytoplasmic transport mechanism in depth, and identifies the core scaffold as a platform for the substantial evolution and adaptation among different species.

IRC as the fixed point for other NPC frame structures and assembled by NUP205, NUP188, NUP155, NUP135, NUP93, and NUP88, is sandwiched by two ORCs [4]. The IRC with two identical rings are located on the equatorial plane of the NPC equator forming links with membrane rings. Recently, 3D maps obtained by cryo-electron tomography have shown a large radial dilation of the IRC in budding yeast (*Saccharomyces cerevisiae*), coupled with a larger central channel and an expanded nuclear pore [16]. Therefore, ORC and IRC act as a scaffold for fixing the core structure of NPC, which is pivotal to NPC integrity [30,36,37].

### 2.2. Cytoplasmic Filaments and Nuclear Baskets

Two characteristic and typical filamentous structures, namely cytoplasmic filaments and nuclear baskets, are asymmetrically distributed on the surfaces of the NPC. These structures are of great significance for specific interactions, serving as docking hubs when transporting biological cargos. The cytoplasmic filaments, which are elongated and structurally disordered polypeptides, have important functions in NPC-capturing cargos. They are mainly composed of NUP214, LOS4, Gle1, CG1, and Rae1 [4]. Moreover, the cytoplasmic filaments play an important role in facilitating the hydrolysis of RanGTP and seem to exclude ribosomes from the space immediately surrounding the nuclear pore [38].

The nuclear basket is constructed of eight protein filaments that are emitted from the NPC core scaffold and join a distal ring into the nucleocytoplasmic side [20]. Its members include NUA, NUP1, NUP50, and NUP82 [4]. Each nuclear basket complex consists of one core architectural and organizational component (in plants this is NUA) which contains a coiled-coil structural domain, and two FG NUPs (NUP136 and NUP50), which bind to the nuclear coaxial ring to connect the NPC core scaffold [39]. Using the proximity labelling-coupled label-free quantitative mass spectrometry (LFQMS) method with NUP82 as bait, a series of nucleoskeleton and nuclear basket were identified. This finding shows that the nuclear basket proteins and the nucleoskeleton are closely interrelated in space [40]. However, most NUPs have no ability to bind directly to DNA or chromatin [41], suggesting that the nucleoskeleton may play a bridge role in nuclear basket involved in regulating gene transcription. With the exception of gene-expression regulation, the NUPs of the nuclear baskets also participate in microRNA biogenesis and transports. For example, NUP1 interacts and colocalizes with THP1 (the core subunits of TREX-2) on the NE to stimulate the export of miRNAs and ARGONAUTE1 (AGO1) from the nucleus to the cytoplasm [42].

### 2.3. Membrane Ring

The membrane ring is the least conserved of all NPC components and displays considerable flexibility among different species; it includes 3–4 transmembrane nucleoporins. The membrane ring anchors the core components of NPC to the pore membrane via a direct interaction with the core scaffold. The main component of the membrane ring in yeast is Pom152 [43]. Glycoprotein 210 acts as the largest component of membrane ring in vertebrate and is homologous with the yeast Pom152, and is involved in regulating cell differentiation [44]. To date, there are only two conserved membrane ring components that have been found in vertebrates, yeast, and plants, namely Nuclear Division Cycle 1 (NDC1) and Glycoprotein 210 (GP210) [25]. Recently, CONSTITUTIVE EXPRESSION OF PATHOGENESIS-RELATED GENE 5 (CPR5) is identified as a new plant transmembrane nucleoporin and is related to disease resistance in *Arabidopsis* [45].

### 2.4. Central FG Nucleoporin

FG nucleoporins account for one-third of all NUPs and are highly conserved among plants and animals. The central FG nucleoporins anchor to the NPC via linker nucleoporins to extend to the cytoplasmic and nuclear sides, and they constitute a central selective channel [46]. The central selective channel acts as gatekeeper to regulate the transport of macromolecules. At present, there are two main hypotheses regarding the selective barrier regulating the transport of macromolecules, namely “selective phase” model and “brush” model [47].

In plants, central FG nucleoporins contain NUP98 and a heterotrimer complex (NUP54-NUP58-NUP62). High speed atomic force microscopy (HS-AFM) results show that they form a liquid–liquid phase separated (LLPS) state to sort cargo transport [48,49], and they use their FG domains to interact with cargo-binding karyopherins that mediate the selective transport of biological cargos larger than around 5 nm or 40 kDa [50]. Selective transport across the central selective channel requires the assistance of nuclear transport receptors (NTRs) [51]. Their efficient transport relies on hydrophobic and electrostatic interactions between FG nucleoporins and NTRs [52]. NTRs can be divided into two groups, importins and exportins. Importins are grouped into importin-α (IMP-α) and importin-β (IMP-β). IMP-α identifies the nuclear localization signal (NLS), and interacts with IMP-β by the B-Binding (IBB) domain for the formation of a heterotrimer (cargo/IMP-α/IMP-β). Subsequently, the heterotrimer complex interacts with central FG nucleoporins to shuttle across the central selective channel. In addition, exportins play a reverse role with importins, which can recognize nuclear export signals (NESs) to facilitate macromolecule export [53]. Among the NTRs identified in human cells, 10 of them are importins, five of them are exportins, three of them are biportins [54]. However, there are still many that are as of yet unknown in kingdom of plants, and the dynamics of central FG nucleoporins are still largely unknown, as central FG nucleoporins LLPS have evaded structural characterization [55].

## 3. Defensive Roles of NPC in Plants

Different NUP mutants represent distinct phenotypes, and different NUPs are found to be involved in the regulation of distinct plant physiological processes, including defensive responses [56,57,58]. These phenomena imply that in addition to nucleocytoplasmic transport, nucleoporins may have other unexpected functions. Meanwhile, there is increasing evidence that NPC contributes to the spatial structure of chromatin, genome integrity, and gene expression pattern, all of which are carefully controlled by different NUPs, independently of their basic transport functions [59]. It has been shown that the different components of NPC are conducive to regulating distinct physiological processes, including main four aspects: immune defense, growth and development, hormone signaling, and temperature response (summarized in Figure 1 and Table 1).

### 3.1. Immune Defense

To antagonize pathogens, plants have evolved a set of immune response strategies. Of these, it is important to regulate nucleocytoplasmic bidirectional transport in immune signal molecules [25]. Using forward genetic screening, a series of nucleoporins participating in immune defense and programmed cell death (PCD) are identified and characterized [60]. For instance, BRI1 Associated Kinase 1 (BAK1) is the co-receptor of Flagellin Sensitive 2 (FLS2) and EF-Tu Receptor (EFR). Mutations in nucleoporins such as *nup85*, *nup96*, *nup160* and *seh1* can suppress *bak1* *bkk1* double-mutant induced PCD [61]. Consistent with this result, NUP96, NUP160 and Seh1 participate in basal immune defense against the bacterial pathogen *Pseudomonas syringae*. Coincidentally, these three NUPs all belong to ORC and mediate mRNA export from the nucleus, suggesting a sophisticated relationship between nucleocytoplasmic transport and immune response [57].

Another NTR, KA120 (IMP-β family), is involved in immune responses by regulating nucleocytoplasmic distribution. The mutation in *KA120* displays resistance to powdery mildew pathogen; in contrast, the overexpression of *KA120* efficiently suppresses the autoimmune phenotype of *snc1-1*. Furthermore, KA120 alters the nucleus distribution and protein activity of SNC1 [62]. In the IMP-α family, IMP-α3 plays a role in defense against *P. syringae*. IMP-α3 is the main receptor of SNC1 among the nine members of the IMP-α family, and IMP-α3 directly interacts with SNC1 at the protein level both in vivo and in vitro [63]. Beyond bacterial and fungal immune defense, NTRs also participate in virus defense. The virulence factor of cucumber mosaic virus (CMV) 2b protein could bind to the host’s AGOs to weaken the immune response. The phosphorylated state of S40, S42, S28 affects 2b and is recognized by IMP-α for importing into the nucleus and then regulating 2b’s nuclear-cytoplasm distribution [64]. Different NTRs combine distinct molecular cargos and play different roles in response to multiple pathogens and dynamic environmental stimuli. However, the precise regulation mechanisms need to be further explored.

The circadian clock modulates the immune response in plants [65]. The mutation in core scaffold nucleoporin IRC NUP205 results in both a defect in the circadian clock and defective immune responses and is accompanied by changes in the nuclear accumulation level of mRNAs related to circadian rhythm and immune defense. Bacterial infection attenuates the expression of clock genes, as in *nup205*. This research indicates that after bacterial infection, transcriptional reprogramming requires integrity and a fully functional NPC, which the circadian clock is at least in part responsible for [65].

Furthermore, CPR5, a novel transmembrane nucleoporin, plays a key inhibitory role in plant ETI. Under normal conditions, CPR5 can trigger homo-oligomerization to restrict the nuclear transport of immune signal cargo; with the NB-LRR receptors activated, CPR5 undergoes a conformation transition from oligomer to monomer, and then enhances NPC permeability, causes an influx of immune-related signaling molecules, and finally stimulates a variety of downstream stress-related signaling pathways [45]. Meanwhile, mutations in *NUP54* and *NUP58* enhance the autoimmune phenotype in *cpr5*; in contrast, the autoimmunity of *cpr5* could be suppressed by the mutation of *nup88* [45] by attenuating the nuclear export of some immune-related proteins, such as EDS1 and NPR1 [66]. Moreover, the loss-of-function of EXPORTIN4 (XPO4) enhances the autoimmunity of *cpr5* by counteracting the nuclear accumulation of TOPLESS (TPL) and TPL related (TPR) proteins; XPO4 coordinates the nuclear accumulation of TPL/TPRs, which co-repress the immune negative regulators and are redundantly required for ETI induction [67]. These examples suggest that nuclear protein influx and efflux are regulated by distinct NPC constituents to mount a robust immune response. In addition, CPR5 is a component of the RNA processing complex (Transformer 2 subfamily in the SR superfamily) and coordinates with NTC and CPSF in plant immunity. Furthermore, CPR5 is the first transmembrane nucleoporin identified in the SR superfamily. Meanwhile, data from the *cpr5* RNA-sequencing indicate that CPR5 participates in the regulation of alternative splicing (AS). Hence, CPR5 may act as a component of LLPS [68] which is involved in the plant immune response [69]. Collaboratively, NUP54, NUP58, NUP88, and XPO4 cooperate with CPR5 to regulate the disease resistance, highlighting that the integrity of the NPC plays pivotal roles in immune signals.

The *HOS1* transcript in peaches (*Prunus persica*) is upregulated in response to priming resistance to *Rhizopus stolonifer* due to a physical interaction with PpWRKY22 in vivo [70]. In *Lotus japonicas*, a defect in *GLE1* slows the symbiotic association with rhizobia, whereas the nitrogen fixation capacities of the *gle1* nodules are far below the wild type, indicating that *GLE1* is critical for the mutualistic symbiosis between legumes and rhizobia [71]. This confirms the significant role of NPC responses to symbiotic and pathogenic microorganisms in leguminous plants.

Salicylic acid plays an absolutely necessary role in plant defense against pathogens. Some NUPs are involved in salicylic acid-dependent immune responses. Single *nup82* mutant has no obvious differences from the wild type, but the *nup82 nup136* double mutant shows severe growth defects and impaired benzothiadiazole (an analog of salicylic acid) induced resistance to *P.* *syringae* [72]. This study shows that there is redundancy in the functions of NUP136 and NUP82. The compromised immune function of the *nup82 nup136* double mutant due to the integrity of the nuclear basket is destroyed; and their deletion triggers an immune defect, in the same way as the *nua* (another nuclear basket protein) mutant, which highlights their collaborative role in maintaining the integrity of NPC and the plant immune response. Furthermore, NUP136 interacts with the Transcription Export 2 (TREX-2) complex and boosts miRNAs and AGO1 export [42], both of which influence the expression patterns of some broad spectrum immune defensive genes [73,74]. The function of three nuclear basket nucleoporins, NUP82, NUP136 and NUA in defensive pathway implies that the nuclear basket is particularly sensitive to the fluctuation of salicylic acid signal.

Pathogen-responsive mitogen-activated protein kinase (MAPK/MPK) cascades are extremely important for activating immune receptors, and then downstream target genes [75]. Furthermore, *nup88* is hypersensitive to *Botrytis cinerea* with reduced activated MPK3 and MPK6 levels. It is possible that the loss-of-function of NUP88 promotes the nuclear export of MPK3 and thus jeopardizes its nuclear accumulation, resulting in compromised resistance to fungi [76]. Furthermore, MPK6 targets the phosphorylated sites VPSSTPLIK(s)*PVATTQQLPK and AP(s)*PGGGSSTIVTLADR of NUA;. The diminished single and dual phosphorylation of SPEKEEVQPETLA(t)*PTQ(s)*PSR of NUA in *mpk3* and *mpk6* mutants [77] reduce resistance to the bacterial pathogen *P. syringae* [25]. Nevertheless, the dependence of this phenomenon on the phosphorylation site of MPK6 requires further evidence.

Another central FG nucleoporin, NUP98, resists necrotrophic fungal pathogen in an NUP88-dependent manner. Indeed, the *nup98ab* double mutant is more susceptible to *B. cinerea* infection [76], while NUP88 interacts with NUP98A and NUP98B to affect the permeability of NPC for the transportation of some immune-related cargos. As with rice, the knockdown of *APIP12* (AvrPiz-t interacting protein 12, homolog of *AtNUP98*) causes an enhanced susceptibility to *Magnaporthe oryzae* by suppressing the expression of pathogenesis related (PR) genes [78]. It is worth noting that NUP54, NUP58, and NUP98, all belong to the central selective barrier, but exhibit opposite immune responses to different fungal pathogen, indicating that different FG-NUPs are distinct in framing the selective channel and in sorting biological cargos [2]. The opposite phenotype caused by a different component of NPC may imply that the two of them feature entirely different mechanisms in disease resistance.

### 3.2. Plant Growth and Development

Early flowering is used as a drought-escape mechanism and late flowering is a cold-response mechanism in plants. Previous studies have shown that NUPs function in the regulation of flowering, implying that NUPs may act against harsh environments by changing flowering time. Some *nup* mutants, such as *nup160*, *nup96*, *hos1*, and *nup98*, show early flowering in *Arabidopsis*. NUP160 and NUP96 stabilize HOS1 protein, a negative regulator of cold, to negatively regulate flower time via ubiquitination and subsequent 26s proteasome degradation of CONSTANT (CO). Coincidentally, NUP160, NUP96 and HOS1 all belong to the ORC component, and function in the regulation of flowering through controlling CO protein level [79,80].

However, with the exception flowering time relying on CO, two redundant nucleoporins NUP98A and NUP98B bypass the CO checkpoint in photoperiodic signaling and integrated signals from many other pathways to take FT as a direct target for flowering control [81]. The *nup98ab* double mutant accumulates more starch and demonstrates a much earlier senescence phenotype compared to control plants, indicating that abnormalities in starch degradation and energy metabolism are the main cause of earlier senescence in *nup98ab* [82]. In addition to participating in the control of flowering, NUP98 positively regulates hypocotyl elongation in response to shade avoidance syndrome (SAS), by modulating shade-induced gene expression in a transport independent manner [83].

In *Arabidopsis*, NUA negatively mediates flowering under both short and long day conditions. NUA recruits and interacts with EARLY IN SHORT DAYS 4 (ESD4), a SUMO protease, which is located at the nuclear rim, to change the accumulation of SUMO conjugates to control flowering [84]. This indicates that NUA functions not only in mRNA export, but also sumoylation [84]. In human, biallelic variants in TPR (NUA in plants) cause microcephaly, ataxia and severe intellectual disability, accompanied by a decrease in nucleus global RNA intensity [85]. This cognition regarding the nuclear basket shows that beyond providing anchors for mRNA transport, it has another mechanism involved in regulating transcription. TPR directly interacts with GANP, a component of the TREX-2 complex [86], demonstrating that TREX-2 complex associate tightly with the NPC in a TPR dependent manner. Furthermore, the maintenance of memory gene loops (MGLs) could lead genes to re-induce quickly and facilitate mRNA accumulation during short term periods of transcriptional repression. Myosin-like protein 1 (Mlp1), a homolog gene of plant NUA in yeast, is important for assembling and maintaining the MGLs. Furthermore. Mlp1 tethers MGLs to the NPC by interacting with MGLs. These two completely different regulatory mechanisms of NUA provide new views for the regulatory mechanism of NUA in plants [87,88].

The *nup160*, *nup96* and *nua* mutants all exhibit early flowering and accompany nuclear mRNA accumulation; *nup85* and *seh1* also exhibit nucleus accumulation of mRNA, however, with no significant flowering phenotype [25]. Therefore, distinct NUPs may be responsible for transporting entirely different sub-pools of mRNAs, or may have other functions independent to mRNA transport, thus resulting in particular phenotypes. Moreover, disturbing the function of plant NUPs causes a significant developmental defect phenotype. The *hos1* mutant exhibits a longer hypocotyl phenotype in light conditions but a shorter one in dark conditions relative to control, similar as in auxin overproducing mutants [89]. Additionally, HOS1 can directly interact with PHYTOCHROME INTERACTING FACTOR4 (PIF4) to participate in phytochrome B (phyB)-mediated hypocotyl elongation [90,91]. The light signal mediated by phytochrome induces the recruitment of light-related genes to the nuclear periphery during transcriptional activation. The heterologous expression of *AtNUP62* in tobacco leads to leaf tissue damage, indicating that the function of AtNUP62 is to maintain tissue integrity [92].

The gametogenesis of male and female is a complex biological process that requires a coordinated expression of genes to regulate the cell proliferation and differentiation. NPC are essential cellular structures that assemble during cell proliferation and differentiation, and transport extensive material cargos for this process. Thus, the mutants of several NPC components have been found to affect embryonic development. Arabidopsis *NUP1* participate in pollen and ovule development. The *nup1* mutant causes defects in both male gametogenesis and female gametogenesis fertility. Most ovules of *nup1-4* stagnate during meiosis, and approximately only 3% of ovules develop to the FG7 stage; the development of pollen grains is aborted during the first mitotic division. Relative to the wild type, the expression of genes related to megasporogenesis and pollen development are significantly down regulated in *nup1-4* mutant [93]. The restriction of enzyme-mediated chromatin immunoprecipitation (RE-ChIP) assays, uses NUP1-GFP as bait and NUP1 is primarily associated with the transcriptionally repressed chromatin region [94]. It is possible that NUP1/136 directly mediates the gene transcription by recruiting transcriptionally repressed chromatin regions to the NPC [93]. Loss-of-function of NUA also produces defects in gametogenesis by disturbing miRNA and mRNA exports [42,84]. Furthermore, NUP88 participates in gametophyte formation during mitosis in *Arabidopsis*, and *nup88* displays a failure of spindle and microtubule formation, a fate specification of gamete cells, impairs ovule and pollen development, and eventually leads to seed abortion [95]. In addition to NUP1, NUA and NUP88, LNO1 is also required for plant embryogenesis and seed viability. As is known, LNO1 is a FG-nucleoporin that is homologous with human NUP214 and yeast NUP159. Homozygous *lno1* is lethal, and the seed viability of heterozygous mutant is aborted. The deletion of LNO1 abolishes asymmetrical cell division during the first zygotic formation, and alters the planes and number of cell divisions in early embryogenesis, which eventually leads to abnormal embryogenesis and embryo abortion [96]. Whether these functions are modulated by specific cell signaling transductions in light of macromolecular transport or transport-independent functions is a challenge to be addressed in the near future.

### 3.3. Plant Hormone Signaling

As reported, some phytohormone signaling pathways feature unique sensitivity to disturbance in plant NPC. Auxin is the main naturally occurring phytohormone that coordinates and steers cell growth and organ differentiation. Furthermore, *NUP160*, *NUP96*, and *NUA* participate in the auxin response, because corresponding nucleoporin mutants are screened as suppressors of the *auxin-resistant1* (*axr1*) mutant [97]. Additionally, NUP62 also plays an important role in the auxin response. The *nup62-1* mutant displays small cotyledons, reduced leaf blades, malformed siliques, and earlier bolting than wild type, while *nup62* mutants are hypersensitive to auxin at the root and cotyledon levels. Furthermore, a mutation in NUP62 leads to an increased activity in the DR5 synthetic promoter. This suggests that NUP62 is a major negative regulator that participates in the auxin signaling response or transportation [97]. Loss-of-function of *TRANSCURVATA 1* (*AtTCU1*, the homologous gene of *NUP58*) shows pleiotropic phenotypes, such as hypocotyl elongation, reduced vegetative leaf size accompanied by a reticulated and greener vein, and early flowering through a direct interaction with auxin signaling pathways [98]. Furthermore, HOS1 negatively regulates auxin biosynthesis in *Arabidopsis* hypocotyl elongation control [89]. In the apple, *MdNUP54* and *MdNUP62* also respond to auxin treatment. Interestingly, 3-Indoleacetic acid (IAA) significantly restrains the expression of seven NUP genes and delays apple flowering.

Nucleoporins also participate in the abscisic acid (ABA) signal response. The *nup85* is identified by a forward genetic screen, which causes *RESPONSIVE TO DESICCATION 29A* (*RD29A*) to reduced expression in response to ABA. After 3 h treatment with 50-µM ABA, several stress related genes including *RD29A*, *COLD REGULATED 15A* (*COR15A*) and *COR4*7 are obviously downregulated in *nup85*. That study demonstrates that *nup160* and *hos1* are sensitive to exogenous ABA as well. Strikingly, a direct internal interaction between NUP85 and Mediator 18 (MED18) is identified by immunoprecipitation combined with mass spectrometry. A direct interaction between NUP85 and MED18 connects the association between NPC components and Mediator core subunits, and suggests the possibility of NUP85 or other NPC components associated with Mediator complex that directly link the regulation of downstream gene transcription mediated by RNA Pol II. However, there is no evidence indicating the member of the NUP107-160 complex directly interacts with and tethers chromatin regions to regulate genes involved in phytohormone signaling [99]. Indeed, auxin and ABA are considered as the two most significantly affected hormones based on *cpr5* transcriptome data [45]. Research on the biological significance of the crosstalk between NPC and plant hormone signaling pathways may yield novel insights into potential regulatory mechanisms.

Furthermore, CPR5 participates in the ethylene signal. It directly interacts with the N-terminal transmembrane domains of ETHYLENE RESPONSE 1 (ETR1), which is the major ethylene receptor in *Arabidopsis*. It can selectively regulate the nucleocytoplasmic transport of mRNAs related to ethylene signaling [100]. Regarding the relationship between NPC and the ethylene signal, there is another classic model that is involved in the tethering of ETHYLENE INSENSITIVE2 (EIN2) to regulate its nucleocytoplasmic transport. Ethylene triggers EIN2 dephosphorylation, cleavages its C-terminal cytosolic region from transmembrane domain, and frees the C-terminal to enter the nucleus to trigger *EIN3* and *EIL1* expression [101,102,103]. It remains to be clarified whether NPC plays a role in this classic model and which component of NPC mediates this process.

### 3.4. Temperature Response

Ambient temperature has a significant influence on plant growth [104], organ development [105] and abiotic or biotic stress responses [106]. Previous studies have indicated that the plant NPC plays important roles in response to temperature cues. The observation of temperature-sensitive defects of some nucleoporin mutants has encouraged more detailed explorations of the role of NPC in perceiving ambient temperature change and in the assessments of how to test speculated mechanisms.

The NUPs of ORC are most frequently reported in the temperature response. Furthermore, NUP160 plays a crucial role in the cold response by impairing the level of cold induced transcription factor CBF3. The *nup160-1* mutant is more sensitive to chilling and features an altered expression of cold relevant genes by impacting the accumulation of mRNA in the nucleus [107]. Furthermore, the *nup96*, *nup160*, and *hos1* mutants all elongate the hypocotyl to respond to high temperatures by suppressing the nuclear accumulation of PHYTOCHROME INTERACTING FACTOR 4 (PIF4), a key factor involved in perceiving temperature and photosynthetic signals. Moreover, the *nup133* and *nup96* mutants show defects in fresh weight after temperature change [108]. As an attenuator of cold stress signaling, HOS1 reduces the expression of cold responsive gene to decrease cold tolerance [109]. Additionally, HOS1, which functions as a RING E3 ubiquitin ligase, directly interacts with Inducer of Cold Expression 1 (ICE1) to mediate the ubiquitination and degradation of ICE1 [110]. Importantly, it responds to cold stress by changing the synthesis of secondary metabolites such as glucosinolates (GSLs), phytoalexins, and flavonol glycosides [111]. These results underscore that different members of ORC may play different roles in responding to different temperatures, indicating the existence of temperature influences on ORC complex integrity and activity [108].

Whether HOS1 is a component of NPC and whether it has nuclear localization are still controversial [112]. The GFP-HOS1 fluorescence signal is detectable in both the nuclear periphery and nucleoplasm, which implies that HOS1 may not be constitutively tethered by NPC [79]. Meanwhile, other proteins such as OPEN STOMATA 1(OST1) [113], BR INSENSITIVE 2 (BIN2) [105] affect the interaction between ICE1 and HOS1 to disturb the ubiquitination and degradation of ICE1 in cold resistance. In addition to its function as an E3 ubiquitin ligase, HOS1 associates with chromatin to regulate gene transcription via its non-proteolytic function [114,115], or binds to NPC to regulate mRNA export [116]. It induces thermo-tolerance by activating DNA repair components in a heat shock protein 90 (HSP90)-dependent manner [117]. In the apple, the expression of *MdNUP62* is significantly upregulated after heat treatments in cultured seedlings of ‘Nagafu No. 2’, transgenic lines that heterologously overexpress *MdNUP62* in *Arabidopsis* and tomato show a significantly reduced tolerance to high temperature [118].

**Table 1:** Summary physiological processes involved in plant NPC.

Related Process	Gene	Location	Organismal Role	Cellular Role	Reference
Immune defense	NUP96	ORC	Basal immune defense against *P. syringae*	Influence nuclear mRNA export	[57,60,61]
	NUP160	ORC	Basal immune defense against *P. syringae*	Influences nuclear mRNA export and protein level of EDS1	[57,60,61]
	Seh1	ORC	Basal immune defense against *P. syringae*	Influences nuclear mRNA export and protein level of EDS1	[57,60,61]
	NUP85	ORC	Basal immune defense against *P. syringae*	Influences nuclear mRNA export and involved in endogenous salicylic acid accumulation	[60,61]
	HOS1	ORC	Related to the resistance to *R. stolonifera*	Interacts with PpWRKY22 in vivo	[70]
	NUP205	IRC	Related to circadian rhythm and immune defense	Influences the nuclear accumulation level of mRNAs related to circadian rhythm and immune defense	[65]
	KA120	IMP-β family	Related to the resistance to powdery mildew pathogen	Alters the nucleus distribution and protein activity of SNC1	[62]
	IMP-α3	IMP-α family	Basal immune defense against *P. syringae*	Interacts with SNC1 at the protein level both in vivo and in vitro	[63]
	IMP-α	IMP-α family	Influences RNA silencing of cucumber mosaic virus 2b protein	Mediates nucleocytoplasmic shuttling of 2b protein	[64]
	XPO4	exportins	Participates in the regulation of CPR5 mediated ETI	Coordinates the nuclear accumulation of TPL/TPRs	[67]
	CPR5	Membrane ring	A key inhibitory role in plant ETI	Participates in transporting of immune signal cargos	[45]
	NUP54	Central channel	Participates in the regulation of CPR5 mediated ETI	Mutation in *nup54* enhance the autoimmune phenotype in *cpr5*	[45]
	NUP58	Central channel	Participates in the regulation of CPR5 mediated ETI	Mutation in *nup58* enhance the autoimmune phenotype in *cpr5*	[45]
	NUP98	Central channel	Related to the resistance to necrotrophic fungal pathogen	Interacts with NUP88 and suppress the expression of pathogenesis related (PR) genes	[76,78]
	NUP82	Nuclear basket	Related to salicylic acid mediated immune response to *P. syringae*	Interacts with NUP136 and RAE1, causes downregulation of immune related genes	[72]
	NUP136	Nuclear basket	Related to salicylic acid mediated immune response to *P. syringae*	Interacts with NUP82, causes downregulation of immune related genes	[72]
	NUA	Nuclear basket	Related to the resistance to *P. syringae*		[25]
	NUP88	Linker	Related to CPR5 and MAPK/MPK mediated immune response	Interacts with NUP98, affect the permeability of NPC and transportation of immune-related cargos	[45,76]
	GLE1	Cytoplasmic flaments	Plays a distinct role in the symbiotic association between legumes and rhizobia	Functions in the export of mRNAs from the nucleus into the cytoplasm	[71]
Plant growth and development	NUP96	ORC	Regulates flowering time and hypocotyl elongation	Interacts with HOS1, affects protein level of CO, critical for nuclear localization of PIF4 protein	[80]
	NUP160	ORC	Regulates flowering time and hypocotyl elongation	Interacts with HOS1, affects protein level of CO, critical for nuclear localization of PIF4 protein	[79]
	NUP98	ORC	Inhibit flowering through clock, photoperiod, and age pathways, regulates hypocotyl elongation	Induces FT expression in a CO independent manner, modulates shade induced genes expression	[81,82,83]
	HOS1	ORC	Regulates flowering time and hypocotyl elongation	Affects protein level of CO, interacts with PIF4 and is critical for nuclear localization of PIF4 protein	[79,80,81,82,83,84,85,86,87,88,89,90,91,112]
	NUA	Nuclear basket	Mediates the flowering under both SD and LD conditions, affects the male and female gametogenesis	Interacts with ESD4, MAD1 and MAD2, affects formation of kinetochores during prophase and prometaphase	[84]
	NUP1	Nuclear basket	Participates in pollen and ovule development	Recruits transcriptionally repressed chromatin regions to the NPC	[93,94]
	NUP62	Central channel	Mediates the growth and development of leaves	Controls of nuclear transport and maintaining tissue integrity	[92]
	NUP88	Linker	Affects the development of ovule and pollen, and eventually leads to seed abortion	Affects spindle and microtubule formation, gamete cells fate specification	[95]
	LONO1	Cytoplasmic flaments	Mediates abnormal embryogenesis and embryo abortion	Abolishes asymmetrical cell division during the first zygotic formation	[96]
hormone signaling	NUP96	ORC	Auxin response	influences nuclear mRNA export, affects the localization of AXR3	[97]
	NUP160	ORC	ABA and auxin response	Influences nuclear mRNA export, affects the localization of AXR3	[97,99]
	HOS1	ORC	ABA and auxin response	Regulates expressions of *YUCs*, *PINs*, *CTR1*, *CYP79B2*	[99]
	NUP85	ORC	ABA response	Interacts with MED18 and regulates expressions of *RD29A*, *COR15A*, *COR4*7	[99]
	NUA	Nuclear basket	Auxin response	Influence nuclear mRNA and miRNA export	[97]
	NUP62	Central channel	Auxin response	Influences the activity of the DR5 auxin responsive promoter	[97]
	NUP58	Central channel	Auxin response	Interacts with SCF (Skp1/Cul1/F-box) ubiquitin ligase complex components	[98]
	CPR5	Membrane ring	ABA and auxin response and ethylene signal	Interacts with ETR1, regulates the nucleocytoplasmic transport of mRNAs related to ABA, auxin and ethylene signaling	[45,100]
Temperature response	NUP160	ORC	Cold and high temperature response	Influence nuclear mRNA export	[107,108]
	NUP96	ORC	High temperature response	Affect the nuclear localization of PIF4 at elevated temperature	[108]
	HOS1	ORC	High temperature response	Affect the nuclear localization of PIF4 at elevated temperature	[108,117]
	NUP133	ORC	High temperature response	Impacts nuclear accumulation of the IAA17 protein at elevated temperature	[108]
	NUP62	Central channel	High temperature response	Interacts with multiple MdHSFs	[118]

## 4. Future Perspective

Plant NPC is considered as an important hub that regulates plant defensive processes. Following the investigation of the phenotype for NUP mutants and the analysis of plant NPC proteome data, significant progress has been made in studies of the NPC structure and biological functions. Some questions on NPCs still require more attention in future studies. (1) Are there other undiscovered proteins as components of NPC? What is the microstructure of plant NPC? The traditional understanding of plant NPC micro-structure is based on insights from yeast and vertebrates. With the progress of microscope technology, it is likely that the plant NPC micro-structure will be resolved in the near future. (2) Can plant NUPs directly bind to chromatin to control gene expression for defense response like animals? What are the effects of the particular spatial location of chromatin to the NPC on gene transcription and epigenetic regulation under harsh conditions? (3) Plant ontogenetic defense against abiotic and biotic stresses has been extensively studied [119,120,121]. Studies have indicated that NPCs functions in plant developmental transitions. Are NPCs involved in the plant ontogenetic defensive pathway? How do NPCs interact with age factors? Additionally, what dynamic changes occur in NPC with plant age or environmental changes? Clarifying the answers to these questions will produce novel insights into the potential regulatory mechanisms of NPCs in plants for agricultural progress and relieve the effects of growing stress factors due to global environmental change.

## Data Availability

Not applicable.
